# Ternary nanocomposite carriers based on organic clay-lipid vesicles as an effective colon-targeted drug delivery system: preparation and in vitro/in vivo characterization

**DOI:** 10.1186/s12951-020-0579-7

**Published:** 2020-01-21

**Authors:** Hyeon Young Kim, Jae Hee Cheon, Sang Hoon Lee, Jeong Youn Min, Seung-Yun Back, Jae Geun Song, Da Hye Kim, Soo-Jeong Lim, Hyo-Kyung Han

**Affiliations:** 1 College of Pharmacy, Dongguk University-Seoul, Dongguk-ro-32, Ilsan-Donggu, Goyang, Korea; 2 Department of Internal Medicine and Institute of Gastroenterology, Yonsei University College of Medicine, Seoul, Korea; 3 Department of Integrated Bioscience and Biotechnology, Sejong University, 209 Neungdong-ro, Gwangjin-gu, Seoul, Korea

**Keywords:** Colon-targeted, Inflammatory disease, Aminoclay, Liposome, Nanocomposite, pH-dependent

## Abstract

This study aimed to develop a new colon-targeted drug delivery system via the preparation of ternary nanocomposite carriers based on organic polymer, aminoclay and lipid vesicles. Budesonide (Bud), an anti-inflammatory drug was chosen as a model drug and encapsulated into three different formulations: liposome (Bud-Lip), aminoclay-coated liposome (AC-Bud-Lip), and Eudragit^®^ S100-aminoclay double coated liposome (EAC-Bud-Lip). The formation of the aminoclay-lipid vesicle nanocomposite was confirmed by energy dispersive X-ray spectrum, transmission electron microscopy, and Fourier-transform infrared spectroscopy. All formulations were produced with a high encapsulation efficiency in a narrow size distribution. Drug release from EAC-Bud-Lip was approximately 10% for 2-h incubation at pH 1.2, implying the minimal drug release in acidic gastric condition. At pH 7.4, EAC-Bud-Lip underwent significant size reduction and exhibited drug release profiles similar to that from AC-Bud-Lip, implying the pH-dependent removal of the outer coating layer. Compared to free Bud solution, EAC-Bud-Lip achieved a higher drug uptake in Caco-2 cells and exhibited a stronger inhibition of TNF-α and IL-6 secretion in LPS-stimulated Raw264.7 cells. Furthermore, a bio-distribution study in mice demonstrated that Eudragit^®^ S100-aminoclay dual coating led to a higher colonic distribution with a longer residence time, which correlated well with the delayed systemic drug exposure in rats. Taken together, the present study suggests that the ternary nanocomposite carrier consisting of Eudragit^®^ S100, aminoclay, and lipid vesicle might be useful as an effective colon-targeted drug delivery system.

## Background

Inflammatory bowel diseases (IBD) are chronic relapsing disorders of the gastrointestinal tract and are caused by complex interactions of environmental and genetic factors along with subsequent changes in immune dysregulation [1, 2]. Ulcerative colitis and Crohn's disease are two major types of IBD, where rectal bleeding, abdominal pain, diarrhea, and weight loss are frequent symptoms [1]. Current medical therapy for IBD attempts to suppress inflammatory episodes, and steroids are frequently prescribed due to their potent anti-inflammatory activity [1, 2]. However, the prolonged use of steroids often leads to toxic side effects associated with their systemic exposure [2–4]. Therefore, targeted local drug delivery to the site of inflammation in the colon should be advantageous in avoiding the undesirable systemic toxicity of steroids and in lowering the dose.

Nano-sized carriers have gained a great attention as a potential colon-specific drug delivery system due to their preferential uptake into inflamed colonic mucosa [5, 6]. Mucosal inflammation causes pathophysiological changes such as disruption of the intestinal barrier, increased mucus production, and the infiltration of immune-related cells, all of which provide an environment favorable for the accumulation of nanoparticles in inflamed regions [5, 6]. Therefore, nanoparticulate drug carriers have been extensively studied for targeting the inflamed intestinal mucosa.

Among nanocarriers, liposomes are promising delivery vectors since they are biocompatible, biodegradable, and capable of encapsulating both hydrophilic and hydrophobic drugs [7, 8]. In addition, liposomes can undergo various surface modifications to control the site of drug release and uptake as well as physiological stability [9–11]. For example, to enhance colonic drug delivery, the liposomal surface can be coated with mucoadhesive or pH-sensitive polymers to release the drug preferably at the distal part of the intestine and also lengthen the retention time in the colon [12–14]. Given that an ideal colon-specific delivery system should prevent premature drug release before reaching the colon, the properties of surface coating materials have a great impact on the effectiveness of liposomal drug carriers for colon targeting.

In the present study, bio-inorganic clay and pH-sensitive polymers were selected as coating materials, and the ternary nanocomposite carrier was constructed based on organic polymer, aminoclay, and lipid vesicles. First, budesonide, an anti-inflammatory drug was selected as a model drug and encapsulated into anionic liposomes. Particularly, krill oil, which is rich in phospholipids decorated with omega-3 polyunsaturated fatty acids, was added into this anionic liposome [15, 16]. Krill oil acts as a biocompatible and water dispersible surfactant, and it also possesses anti-inflammatory properties [17]. Then, the surface of these anionic liposomes was coated with aminoclay (3-aminopropyl functionalized magnesium phyllosilicate), a silicate-based material. Since aminoclay has a positive charge when dispersed in water, it can construct a hybrid nanocomposite with anionic lipid vesicles via electrostatic interactions [18, 19]. Furthermore, aminoclay is biocompatible and may have anti-inflammatory effect [20, 21]. Therefore, lipid vesicles coated with aminoclay may be beneficial in improving the gastrointestinal (GI) stability of liposomes and may have the synergistic effect in anti-inflammation. Subsequently, the lipid vesicle-aminoclay nanocomposite underwent the surface coating with a pH-sensitive methacrylate copolymer (Eudragit^®^ S100). Since Eudragit^®^ S100 dissolves at pH greater than 7.0 [14, 22], a Eudragit^®^ S100 coating layer can be retained in the stomach and upper intestine but will dissolve in the distal intestine. This would allow for the targeted release of the drug in the distal intestine and colon. Such a drug delivery approach could be useful in lowering the therapeutic dose, reducing systemic side effects, and improving the physicochemical properties of drugs for oral delivery.

In the present study, three liposomal formulations of budesonide [uncoated liposome (Bud-Lip), aminoclay-coated liposome (AC-Bud-Lip), and Eudragit^®^ S100/aminoclay-coated liposome (EAC-Bud-Lip)] were prepared by optimizing formulation variables. The in vitro and in vivo effectiveness of these formulations was evaluated and compared with conventional powder formulations.

## Results and discussion

### Preparation and characterization of liposomes

**Fig. 1:**
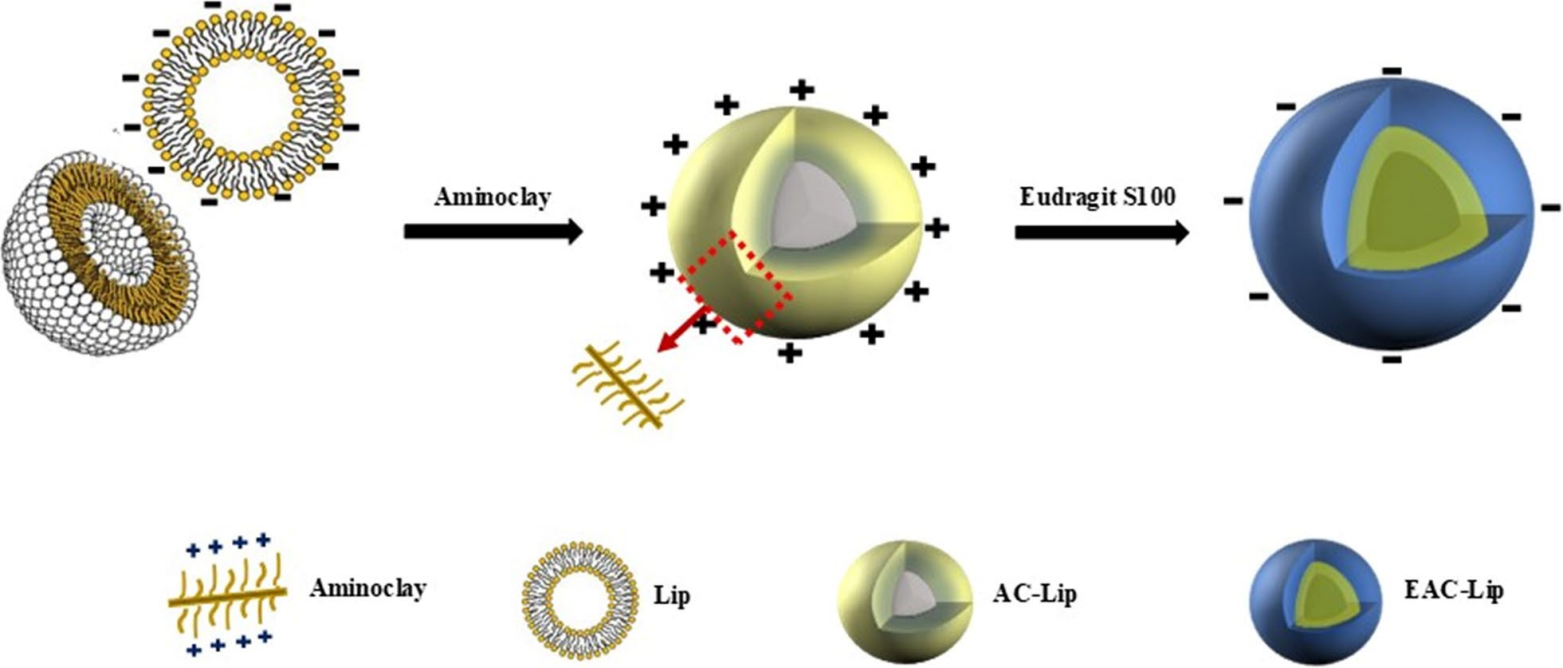
Schematic illustration of organic clay-lipid vesicles

As illustrated in Fig. 1, two oppositely charged polyelectrolytes, aminoclay and Eudragit^®^ S100, were assembled directly on the liposomal surface via a layer by layer deposition to prepare pH-triggered drug releasing carriers for colonic drug delivery. Each formulation was prepared with narrow size distribution and high entrapment efficiency as summarized in Table 1. The net negative surface charge of Bud-Lip facilitated the alternate deposition of the positively charged aminoclay via electrostatic interaction. The addition of aminoclay resulted in a surface charge reversal and an increase in the particle size from 117 to 194 nm. The addition of anionic Eudragit^®^ S100 as a pH-sensitive layer resulted in the alteration of the surface charge to negative and increased the particle size (321 nm) when compared to AC-Bud-Lip. This indicated the successful fabrication of a pH-triggered drug-releasing carrier, EAC-Bud-Lip (Table 1).

**Table 1:** Characteristics of liposomal formulations (Mean ± SD, n = 3)

Formulation	Size (nm)	PDI	Zeta potential (mV)	Encapsulation efficiency (%)
Bud-Lip	117 ± 10	0.42 ± 0.02	− 21.4 ± 0.7	95.6 ± 1.7
AC-Bud-Lip	194 ± 12	0.25 ± 0.11	7.0 ± 2.9	74.7 ± 7.6
EAC-Bud-Lip	321 ± 18	0.40 ± 0.12	− 12.8 ± 5.8	82.7 ± 9.8

**Fig. 2:**
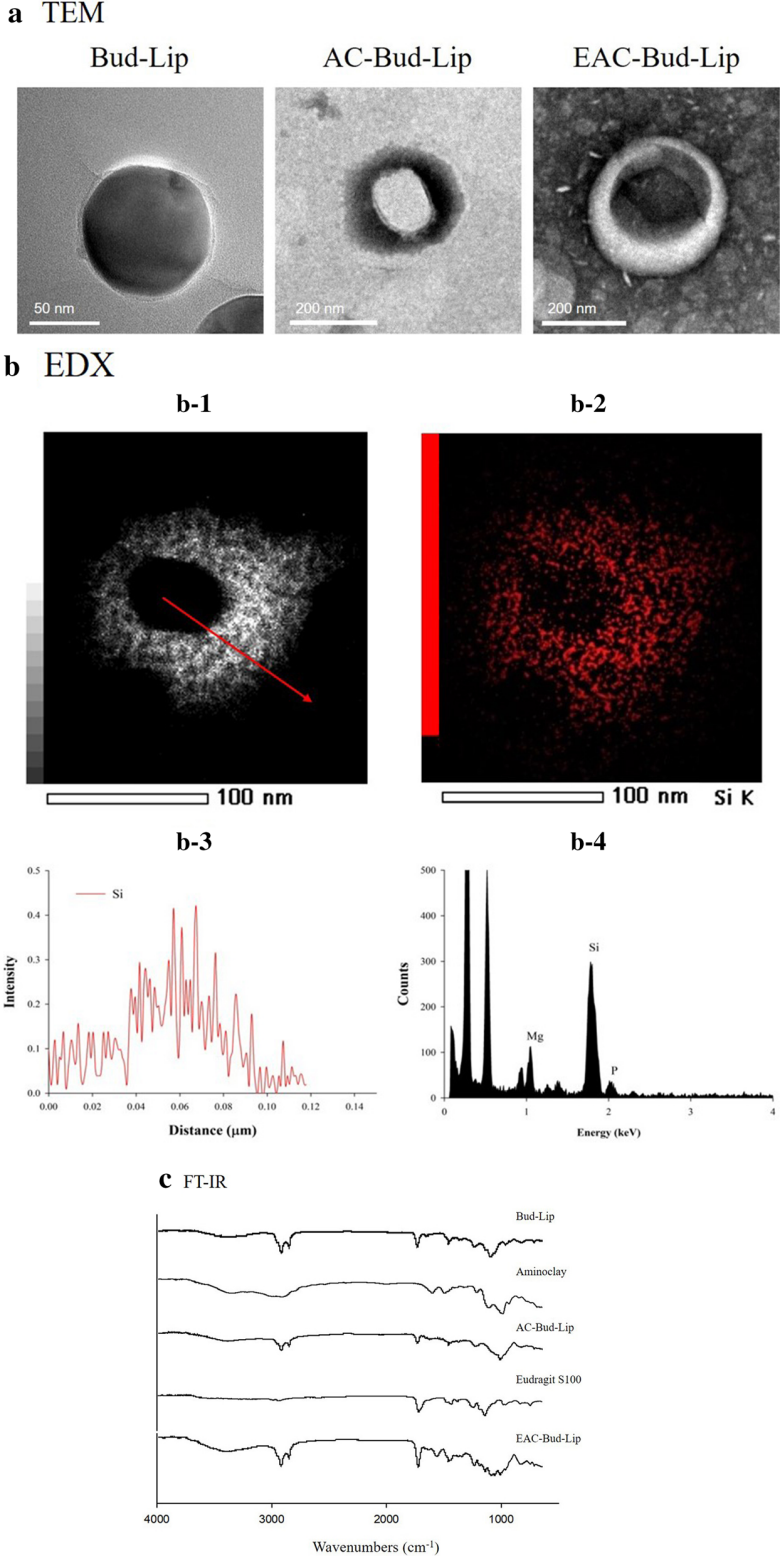
TEM images (**a**), TEM–EDX analysis data of AC-Bud-Lip (**b**), and FT-IR spectra (**c**). EDX line scan analysis data were obtained along the arrow shown in **b-1**

The formation of organic–inorganic nanocomposite carriers was also confirmed by multiple analysis including TEM, EDX, and FT-IR analysis. As shown in Fig. 2a, TEM images showed the spherical shape of the liposomes and the coating layer surrounding the surface of the core liposome. Furthermore, the EDX spectrum of AC-Lip clearly indicated the distinct components originating from the phospholipids (P) and aminoclay (Si, Mg), which confirmed the integration of the lipid and aminoclay components within the electron dense hollow particles (Fig. 2b). EDX mapping and line scan analysis of AC-Bud-Lip were also performed to examine the atomic distribution of Si, a distinct component of aminoclay. As shown in Fig. 2b, the Si component was distributed mainly in the shell surrounding the core liposome, confirming the surface coating with aminoclay. FT-IR analysis further supported the formation of organic–inorganic nanocomposite carriers. As shown in Fig. 2c. FT-IR spectrum of EAC-Bud-Lip indicated the characteristic absorption bands of phospholipids, aminoclay and Eudragit^®^ S100, including a vibrational band of PO_2_− at 1232 cm^−1^, a stretching vibration band of P=O at 1090 cm^−1^, a band of Si–O–Si at 1008 cm^−1^ from the phyllosilicate framework of aminoclay, and bands from Eudragit^®^ S100, C=O at 1730 cm^−1^ and C–O stretch vibration at 1149 cm^−1^ [23–25].

Collectively, the structural characterization confirmed the formation of a ternary nanocomposite carrier based on organic polymer, aminoclay, and lipid vesicles.

### In vitro drug release study

#### Drug release study at different pHs

**Fig. 3:**
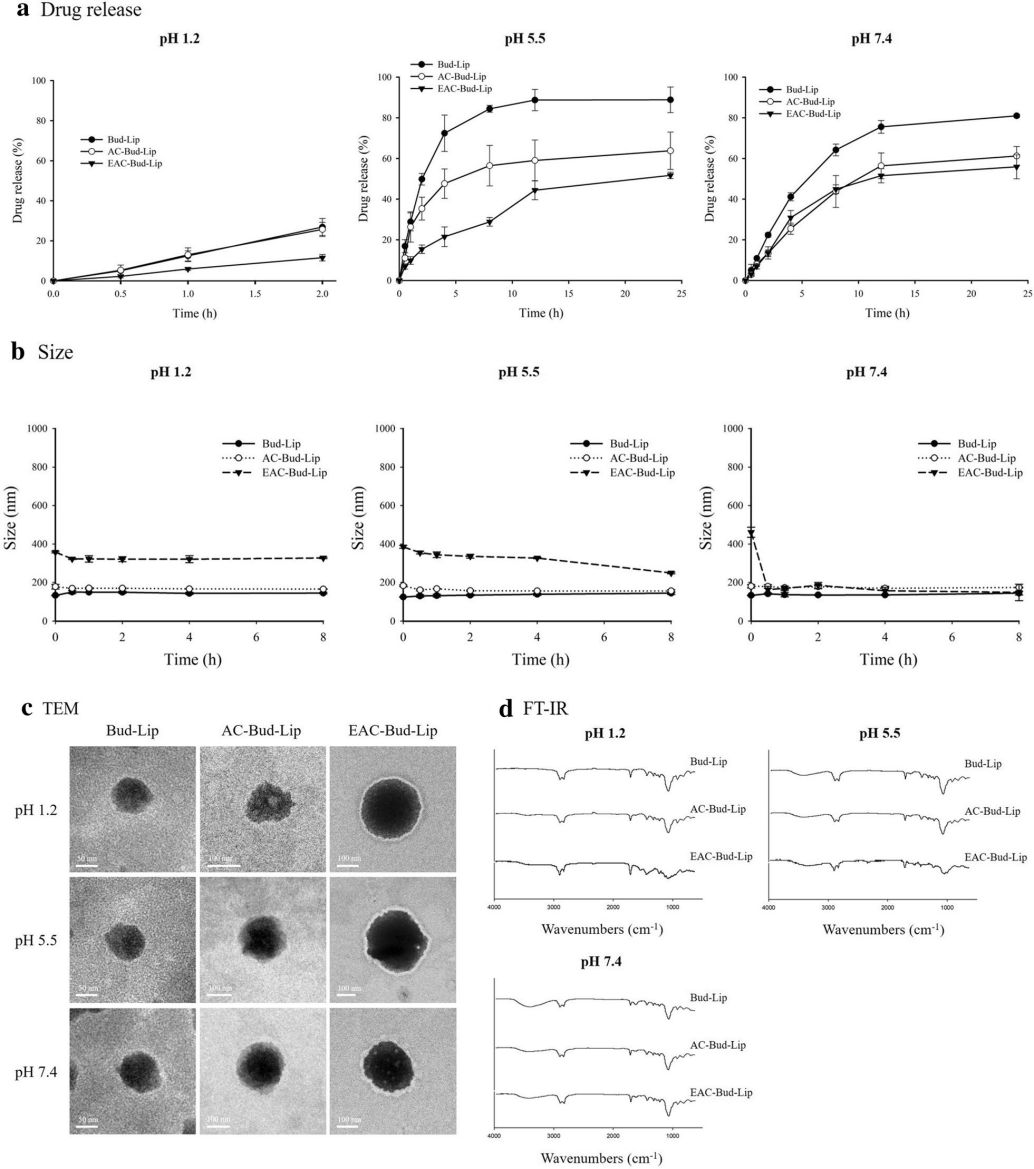
In vitro drug release (**a**), size variation (**b**), TEM (**c**), and FT-IR spectra (**d**) of liposomal formulations at the different pH (Mean ± SD, n = 3). TEM images and FT-IR data were obtained at 2 h-incubation in each drug release medium

Drug release profiles from each liposomal formulation were examined at pH 1.2, 5.5, and 7.4 (Fig. 3a). Among the tested formulations, the delayed and reduced drug release was observed with EAC-Bud-Lip. Furthermore, EAC-Bud-Lip achieved more drug release as the surrounding pH increased, displaying the pH-dependent drug release profiles. Particularly, EAC-Bud-Lip exhibited minimal drug release (~ 10%) at pH 1.2 for 2 h, implying that it could prevent premature drug release in acidic gastric conditions. At pH 7.4, both EAC-Bud-Lip and AC-Bud-Lip showed similar release profiles with approximately 55–60% of release in 24 h, since Eudragit^®^ S100 coating layer could be rapidly dissolved at pH 7.4 (Fig. 3a). This is also supported by the particle size change as illustrated in Fig. 3b. The overall particle size of Bud-Lip and AC-Bud-Lip was retained with minimal variation as the pH changed from acidic to neutral. In the case of EAC-Bud-Lip, its particle size was well maintained at pH 1.2 but rapidly decreased during the incubation at pH 7.4, implying the dissolution of the pH-sensitive Eudragit^®^ S100 coating layer at pH 7.4 (Fig. 3b).

To examine the structural and morphological changes of liposomal formulations during the drug release studies, TEM and FT-IR analysis were also conducted after 2-h incubation in each release medium. As shown in Fig. 3c, TEM images showed that Bud-Lip and AC-Bud-Lip underwent obvious shape change after 2-h incubation at the acidic to neutral pH, along with greater change as pH increased. In the case of EAC-Bud-Lip, it maintained the spherical shape at pH 1.2 but displayed the morphological change at pH 5.5 and pH 7.4, particularly for the surface, which may lead to more drug leakage. In parallel, FT-IR spectra also indicated the structural change in coated liposomes (Fig. 3d). At all tested conditions, FT-IR spectra of AC-Bud-Lip appeared to be similar to those from Bud-Lip along with the disappearance of Si–O–Si band at 1008 cm^−1^, implying the removal of aminoclay coating layer during the incubation. In the case of EAC-Bud-Lip, the distinct absorption bands from aminoclay and Eudragit^®^ S100 were retained in the acidic conditions but disappeared at pH 7.4. This result supported the pH-dependent dissolution of outer coating layer of EAC-Bud-Lip.

Taken together, the results suggest that EAC-Bud-Lip may avoid premature drug release in acidic conditions and effectively retain the entrapped drug during their transit from the stomach until reaching the colon.

#### Drug release study in simulated gastrointestinal fluids

**Fig. 4:**
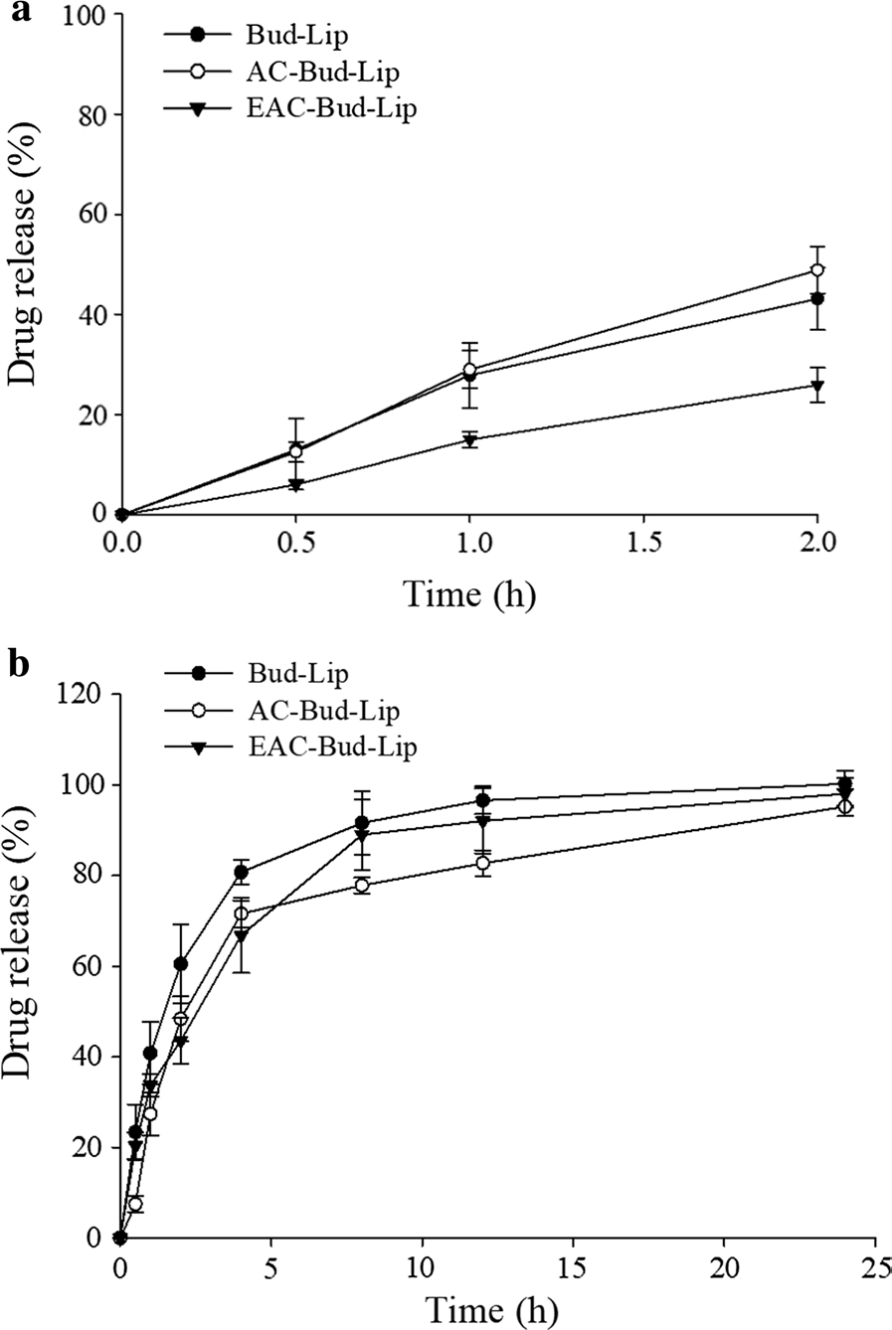
In vitro drug release profiles of liposomal formulations in simulated gastric fluids (**a**) and simulated intestinal fluids (**b**) (Mean ± SD, n = 3)

Since the bile salts and enzymes in the GI tract can accelerate the degradation of liposomal membrane leading to the drug leakage, the drug release profiles were examined in the presence of bile salts and/or digestive enzymes by using the simulated gastric fluids (SGF) and intestinal fluids (SIF). As summarized in Fig. 4, the released drug amount was higher both in SGF and SIF compared to those incubated in buffers at the corresponding pH (Fig. 3a). In addition, drug release rate was enhanced in SIF relatively to SGF, which is also in agreement with the previous findings [26, 27]. These results may be because bile salts and the digestive enzyme (pancreatin) had synergistic effects on the destruction of liposomal membranes in SIF. Particularly, bile salts could damage the liposomal integrity by solubilizing membrane and forming transient pores, accelerating the drug leakage [28].

Collectively, the drug release study in SGF and SIF suggest that EAC-Bud-Lip can prevent the premature drug release during passage through the stomach and release drugs rapidly in the intestinal environment at pH 7.4.

### Cellular uptake study

While normal colonic pH ranges from 6.8 in the proximal colon to 7.2 in the distal colon, colonic pH values for IBD patients significantly vary from pH 5.5 to 2.3 [29, 30]. Therefore, in the present study, the cellular uptake of the developed formulations was assessed at pH 5.5 and pH 7.4 in Caco-2 cells.

**Fig. 5:**
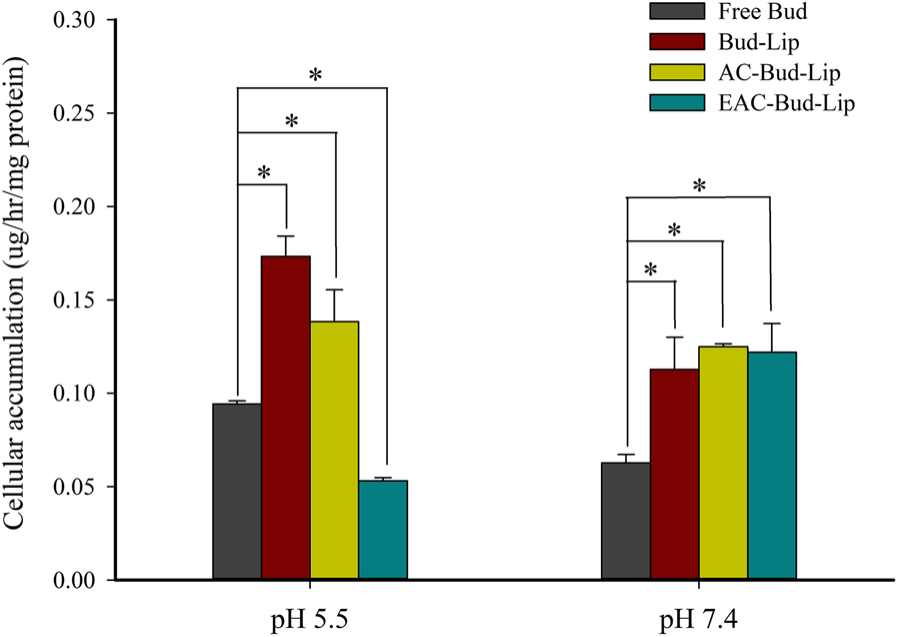
Cellular uptake of budesonide from liposomal formulations in Caco-2 cells at pH 5.5 and pH 7.4 (Mean ± SD, n = 3). *p < 0.05

As shown in Fig. 5, all three liposomal formulations achieved approximatively twofold higher cellular uptake of budesonide than free drug solution at pH 7.4, implying that the liposomal vesicles effectively penetrated the cell membrane to deliver the drug. However, at pH 5.5, EAC-Bud-Lip exhibited significantly lower cellular uptake than free drug solution while Bud-Lip and AC-Bud-Lip still retained their higher cellular uptake. These results could be explained by the pH-triggered dissolution of Eudragit^®^ S100 at pH 7.4. While the outer coating layer of Eudragit^®^ S100 was retained at pH 5.5, it was likely removed at pH 7.4. Several previous studies have also observed a rapid degradation of Eudragit^®^ S100 at pH 7.4 [31, 32]. These results, in conjunction with the in vitro stability data, suggest that EAC-Bud-Lip might minimize drug absorption as well as premature drug release in upper GI tract. Once EAC-Bud-Lip reaches the lower intestine, it can be converted into AC-Bud-Lip, which provides a more favorable cellular uptake even at pH 5.5. Therefore, EAC-Bud-Lip may be able to improve drug delivery to the colon and confer effective cellular uptake in inflamed tissues exhibiting a low pH [33].

**Fig. 6:**
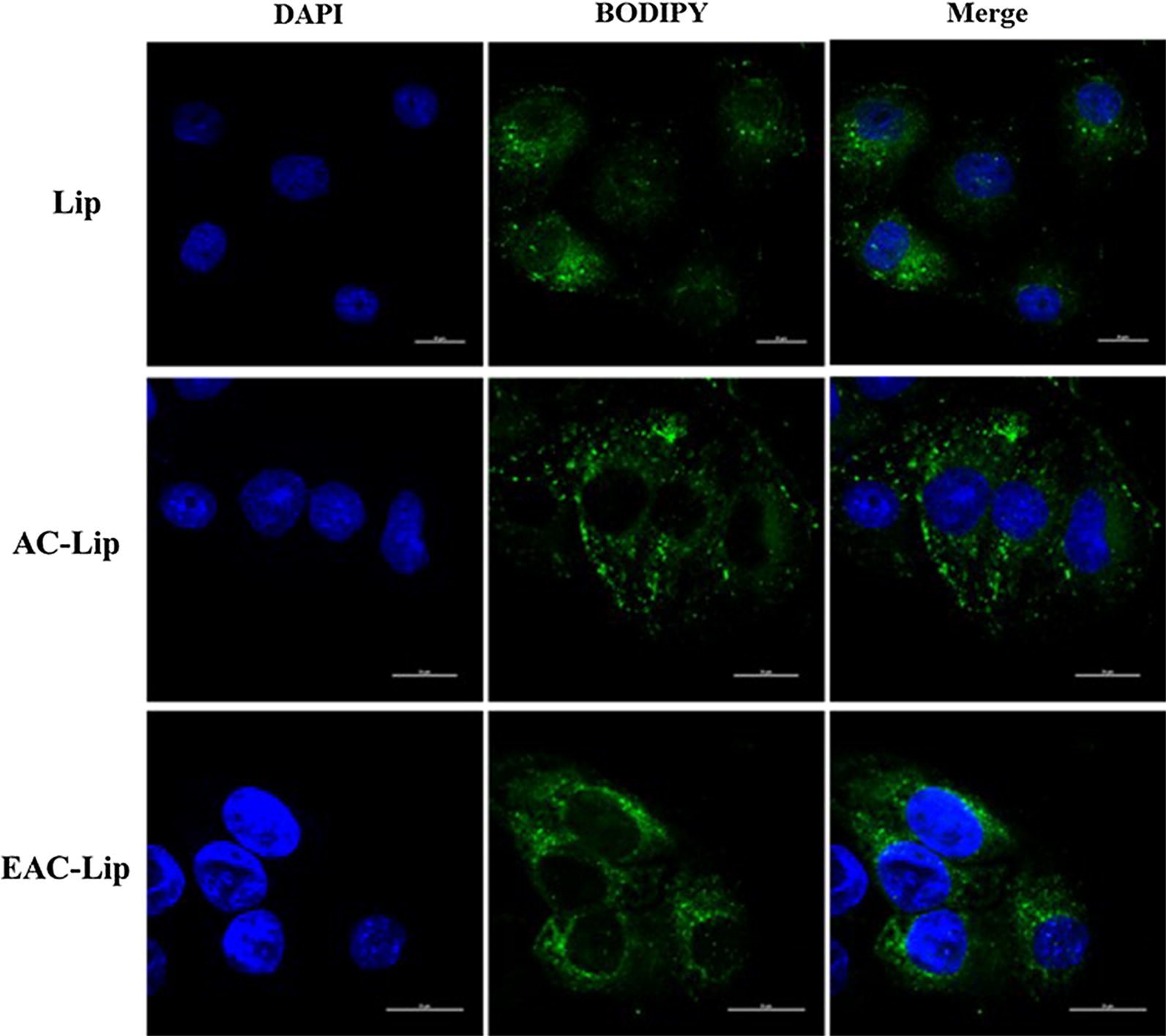
CLSM images showing the intracellular trafficking of bodipy-tagged fluorescent liposomal vesicles in Caco-2 cells. The first panel shows DAPI-stained nuclei, and the second panel displays the green fluorescence related to bodipy. Scale bar is 20 μm

Bio-imaging assay by confocal microscopy also confirmed the effective intracellular distribution of fluorescently labeled liposomes. As shown in Fig. 6, after incubation with each formulation at pH 7.4, strong fluorescence intensity of bodipy-tagged liposomes was observed in the cytoplasm of cells around DAPI-stained nuclei. These results indicate the effective intracellular trafficking of the liposomal formulations. Considering that the particle size is a key parameter in determining the cellular uptake of nanoparticles, the obtained liposomal formulations were in the size range that favors intestinal uptake of the nanoparticles (100–200 nm) [34, 35].

### Anti-inflammatory effect

In IBD, intestinal epithelial cells and macrophages secrete large amounts of chemokines and pro-inflammatory cytokines in the inflamed intestine [36]. Since IL-6, a versatile inflammatory cytokine, is involved in many physiological processes, especially acute and chronic inflammation, it has been used as a standard biomarker of inflammation in comparative studies [37]. TNF-α is also involved in inflammatory and immune responses regulation and it plays an important role in the pathogenesis of IBD [38].

Given that the production of pro-inflammatory cytokines such as TNF-α and IL-6 increase in colon epithelial cells and mucosa of IBD patients [37–39], the anti-inflammatory effects of the developed nanocarriers against the secretion of TNF-α and IL-6 were examined in LPS-stimulated RAW264.7 cells. None of the formulations showed any toxicity to macrophages after 6 h-incubation at the tested concentration (equivalent to 10 µM of budesonide).

**Fig. 7:**
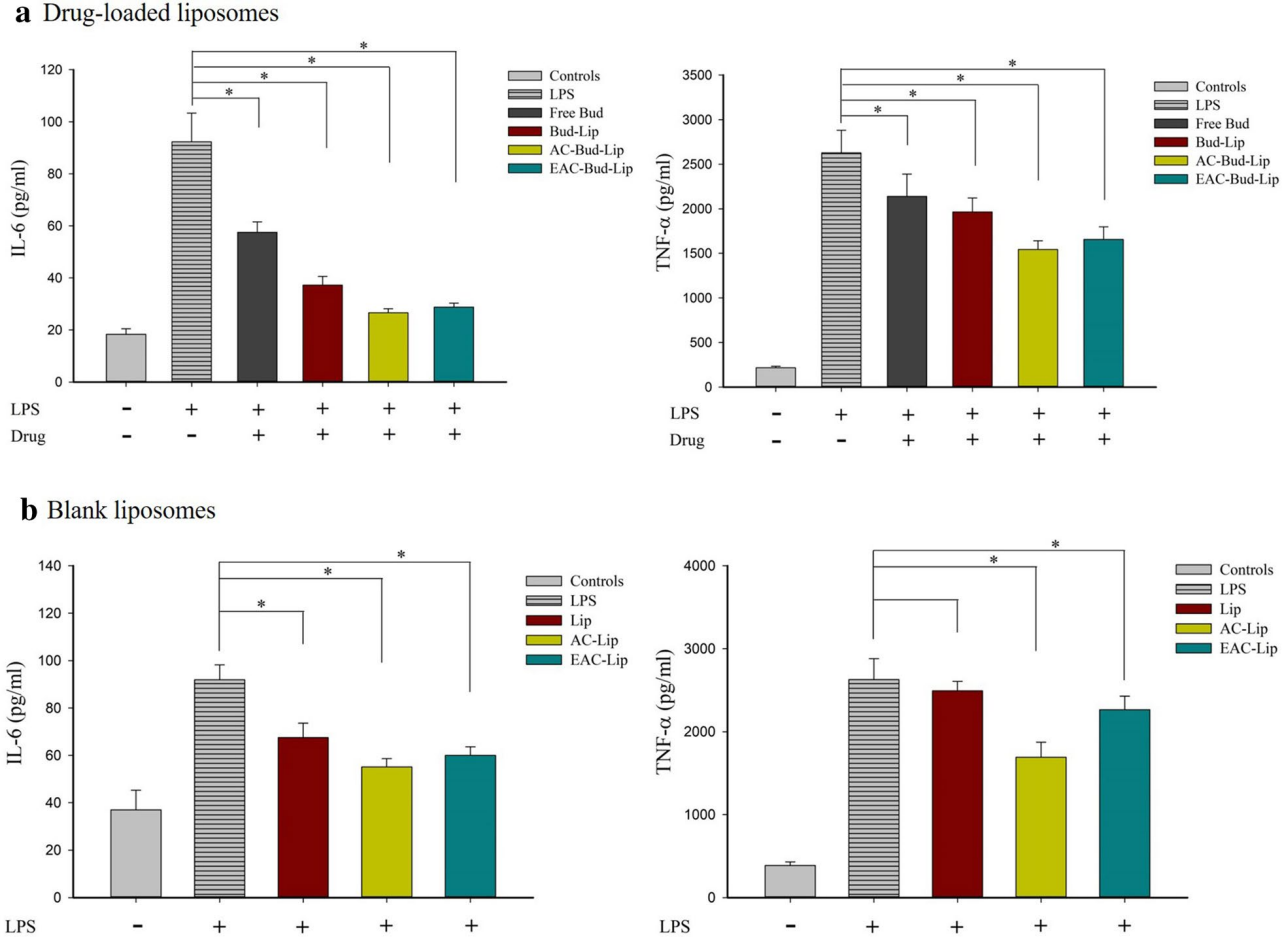
Anti-inflammatory effect of drug-loaded formulations (**a**) and empty vehicles (**b**) in LPS-stimulated Raw264.7 cells (Mean ± SD, n = 6). *p < 0.05

As summarized in Fig. 7a, all formulations significantly reduced LPS-induced production of TNF-α and IL-6. Interestingly, all liposomal formulations enhanced the anti-inflammatory effect of budesonide compared to free drug solution, which may be explained, in part, by the higher cellular uptake of drugs via nanocarriers. Similar levels of inhibition between AC-Bud-Lip and EAC-Bud-Lip could result from the removal of the outer coating layer of EAC-Bud-Lip at pH 7.4. Furthermore, coated liposomes, such as AC-Bud-Lip and EAC-Bud-Lip, exhibited higher inhibition effect than uncoated liposomes, implying that the surface coating with aminoclay may enhance the anti-inflammatory effect of nanocarriers. Therefore, we also tested the inhibition effect of empty carriers on the secretion of TNF-α and IL-6 under the same conditions. As shown in Fig. 7b, empty liposomes without surface coating exhibited an anti-inflammatory effect, which is consistent with previous studies that have observed the immunomodulatory ability of lipid-based nanoparticles [40, 41]. Moreover, the aminoclay-coating further enhanced the anti-inflammatory effect of lipid vesicles. These results suggest that surface coating of lipid vesicles with aminoclay may have a synergistic effect in the anti-inflammatory activity of lipid-based formulations. Consequently, the greatest inhibition effect of coated liposomes on the secretion of TNF-α and IL-6 might be derived from the enhanced cellular uptake of drugs as well as the anti-inflammatory effect of aminoclay-lipid based composite nanocarriers.

### Ex vivo imaging study

**Fig. 8:**
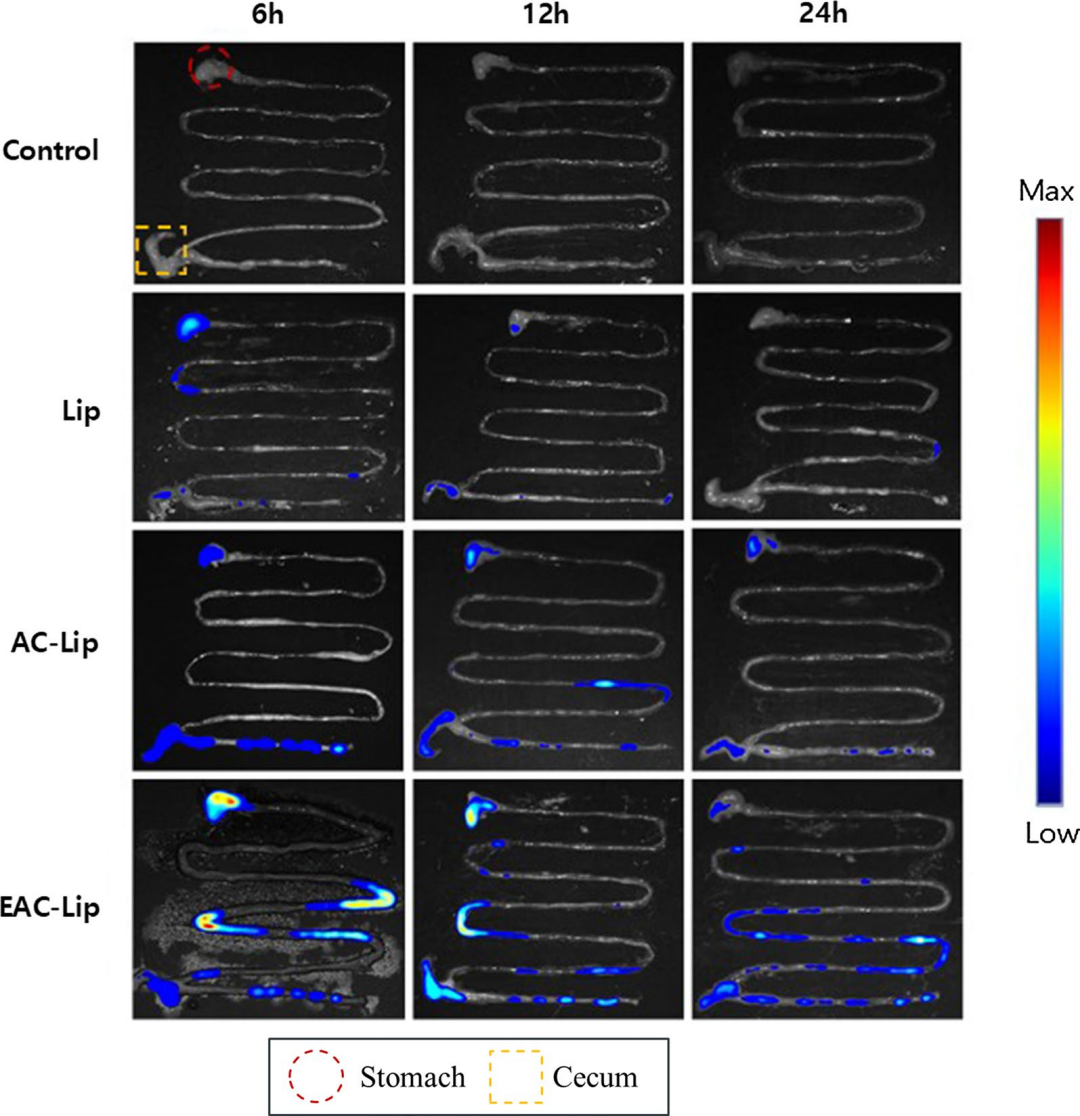
Ex-vivo fluorescence images of the gastrointestinal tract of mice after oral administration of DiR-labeled liposomal formulations

To evaluate the colon-targeted drug delivery potential of the developed nanocarriers, the gastrointestinal distribution of DiR-loaded formulations was examined in mice. After oral administration of each formulation to mice, whole gastrointestinal (GI) tract was removed at 6, 12 and 24 h and the distribution of fluorescent dye was examined by using an in vivo imaging system as shown in Fig. 8.

Blank liposomes were also administered in mice to discriminate the effect of carrier material and no fluorescence signals were observed (Fig. 8). Uncoated liposomes showed low fluorescence signals in GI tracts, probably due to systemic absorption during the transition along the GI tract before reaching the colon (Fig. 8). Particularly, no fluorescence signals were observed in colons at 24 h post-dose of uncoated liposomes. In contrast, at 6 h post-dose, AC-Bud-Lip exhibited strong fluorescence signals mainly in the lower intestine and colon but the fluorescence intensity gradually decreased with time, leading to the absence of fluorescence signals in the lower intestine and colon, which may be due to the colonic absorption or clearance via feces. Among the tested formulations, dual-coated EAC-Bud-Lip exhibited dominant fluorescence signals in the GI tract at all time points, implying that EAC-Bud-Lip may prevent premature drug release and subsequent systemic drug absorption in the upper intestine. Accordingly, compared to other formulations, EAC-Bud-Lip achieved a much greater colonic distribution of fluorescence dye and retained fluorescence signals even after 24 h post-dose (Fig. 8). These results suggest that EAC-Bud-Lip should be more effective in enhancing drug delivery to the colon with longer residence time.

### Pharmacokinetic study

To assess the effectiveness of the developed formulations as local drug delivery systems to reduce systemic effects, budesonide was replaced in each liposomal formulation by coumarin 6 (C6) to facilitate in vivo detection and quantification in the colon after oral administration. Following oral administration of C6 solution or C6-loaded liposomal formulations, systemic exposure and colonic accumulation of C6 was determined in rats.

**Fig. 9:**
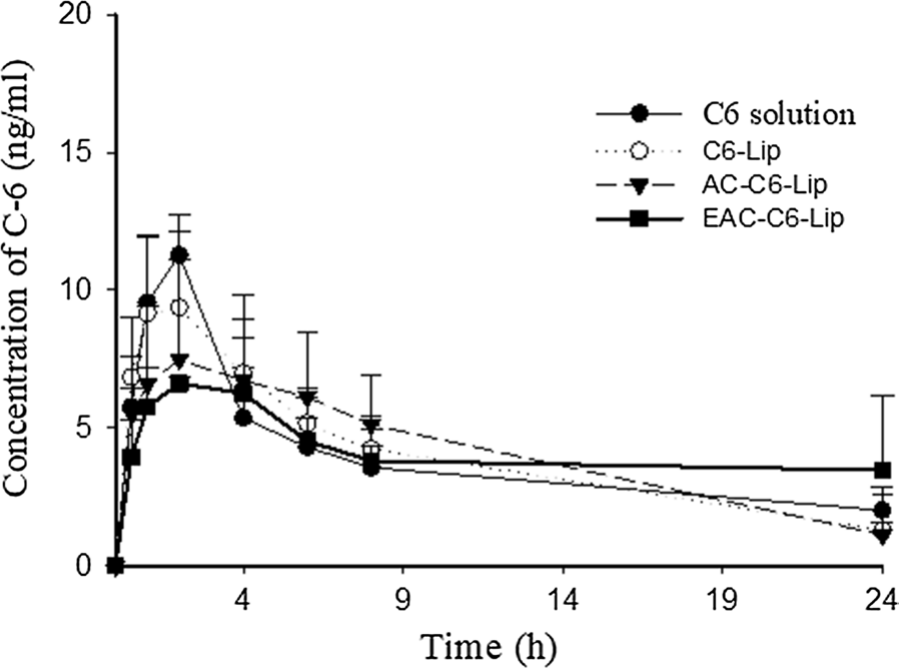
Plasma concentration–time profiles after oral administration of coumarin 6 (C6) in different formulations in rats (Mean ± SD, n = 4)

**Fig. 10:**
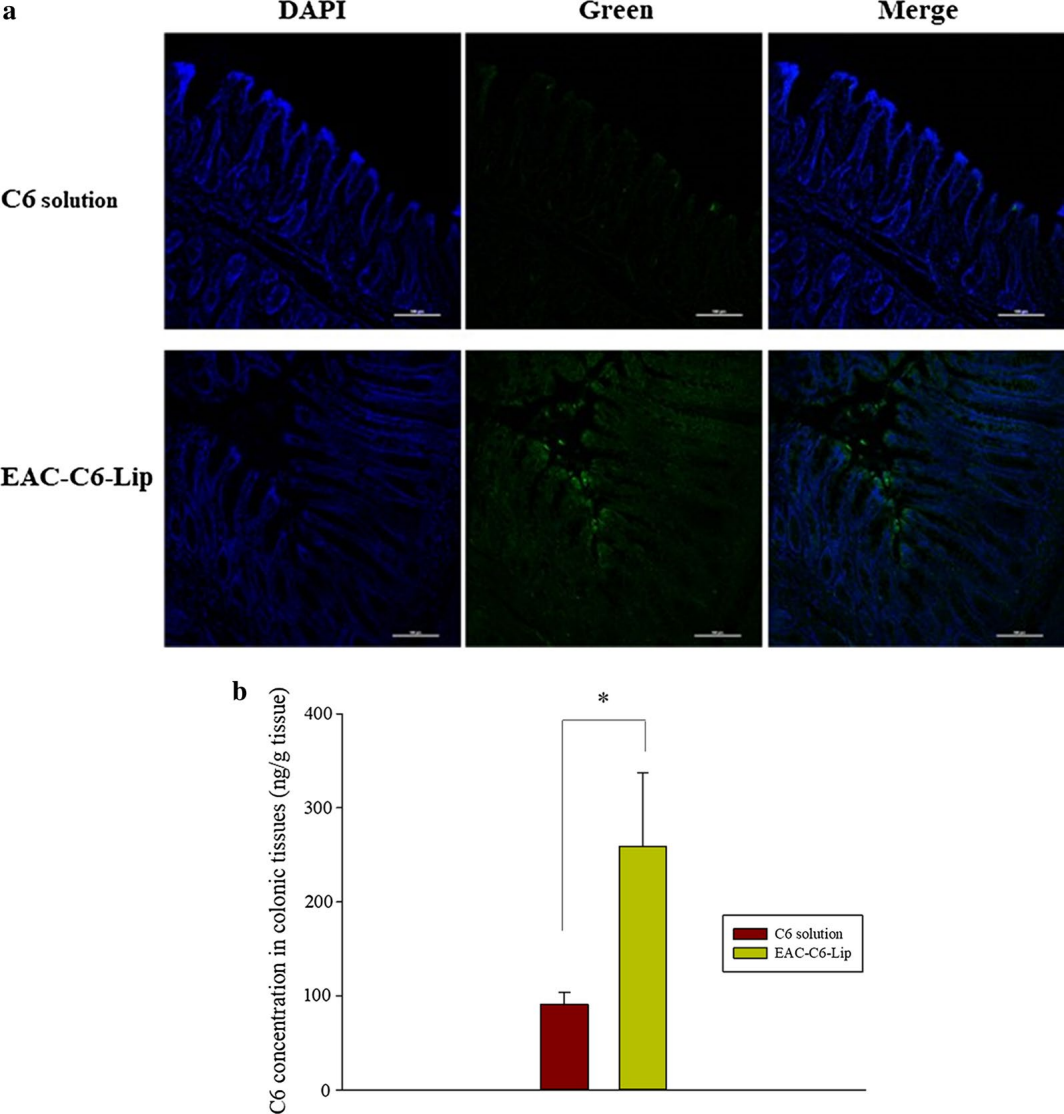
In vivo localization of coumarin 6 (C6) in colons after oral administration of C6-loaded liposomal formulations in rats. **a** Confocal images of C6-loaded liposomes (EAC-C6-Lip) in colon cross sections prepared at 24 h (scale bar is 100 μm), **b** quantitation of C6 in colon tissues at 24 h after oral administration of each formulation (Mean ± SD, n = 3). *p < 0.05

As summarized in Table 2 and Fig. 9, C6 solution and uncoated liposomes (C6-Lip) exhibited rapid absorption with T_max_ values of 1.6–2.0 h after oral administration in rats. In contrast, coated liposomes tended to have longer T_max_ and lower C_max_, implying delayed absorption. Particularly, dual coated liposomes (EAC-C6-Lip) exhibited prolonged drug exposure up to 24 h and C_max_ was reduced by about 35% compared to C6 solution (Table 2), implying that dual coated liposomes may be beneficial in reducing the toxicity associated with unnecessarily high C_max_. In parallel, the colonic tissue distribution of C6 was also evaluated after 24 h following the oral administration of each formulation in rats. As illustrated in Fig. 10a, confocal images revealed that EAC-C6-Lip was spread along the colon tissue, while C6 solution displayed negligible fluorescence intensity. In addition, quantitative fluorescence analysis indicated that EAC-C6-Lip achieved much higher C6 concentrations in colonic tissues than C6 solution, implying more favorable colonic drug delivery (Fig. 10b). Taken together, the observations from the pharmacokinetic study and colonic tissue accumulation study were consistent with gastrointestinal distribution study described above, which strongly suggest more effective colonic drug delivery with longer residence time via double coated liposomes with aminoclay and Eudragit^®^ S100.

**Table 2:** Pharmacokinetic parameters of coumarin 6 (C6) after oral administration of C6 in the different formulations to rats (Mean ± SD, n = 4)

Parameters	Control (C6 solution)	C6-Lip	AC-C6-Lip	EAC-C6-Lip
C_max_ (ng/mL)	11.3 ± 1.46	10.1 ± 2.08	8.0 ± 4.3	7.4 ± 1.3
T_max_ (h)	2.0	1.6 ± 0.5	4.0 ± 2.3	7.8 ± 10.9
AUC (ng h/mL)	91.8 ± 21.0	97.0 ± 18.9	99.7 ± 38.4	99.9 ± 17.7

Given that cationic nanoparticles adhere to the mucosal surface via the interaction between the positively charged nanoparticle and the negatively charged intestinal mucosa, the mucoadhesion properties of cationic nanoparticles can promote better contact with the mucosal surface for cellular uptake and increase drug residence time in the GI tract. Therefore, shielding the cationic surface charge of nanocarriers before reaching the colon and subsequent de-shielding in the colon should be advantageous for prolonged colonic drug delivery. In that sense, a Eudragit^®^ S100 coating layer should be beneficial in preventing premature drug release and undesired mucoadhesion of nanocarriers in the upper GI tract. Once it reaches the distal part of the intestine, the pH-dependent dissolution of Eudragit^®^ S100 releases positively charged AC-Bud-Lip which could interact with negatively charged colonic mucin and reduce the clearance of nanocarriers, leading to longer residence time.

Taken together, pharmacokinetic studies in rats suggest that dual coated liposomal formulations with Eudragit^®^ S100 and aminoclay could be beneficial in increasing local drug delivery to the colon.

## Conclusion

In the present study, three different liposomal formulations were prepared with narrow size distribution and high encapsulation efficiency. Among the developed formulations, EAC-Bud-Lip showed good stability with minimal drug leakage in the acidic conditions and pH-dependent dissolution of coated layer. Furthermore, EAC-Bud-Lip appeared to be effective at enhancing the cellular uptake and the anti-inflammatory effects of budesonide. In bio-distribution and pharmacokinetic studies, EAC-Bud-Lip exhibited higher colonic distribution with a longer residence time as well as lower C_max_. These results suggest that EAC-Bud-Lip could be a promising colon-targeted delivery system.

## Methods

### Materials

Budesonide was obtained from Tokyo Chemical Industry Co., LTD. (Tokyo, Japan). 1,2-dimyristoyl-*sn*-glycero-3-phosphocholine (DMPC), 1,2-dimyristoyl-*sn*-glycero-3-phospho-1′-*rac*-glycerol sodium salt (DMPG), and bodipy-cholesterol were purchased from Avanti Polar Lipids, Inc. (Alabaster, AL, USA). d-α-tocopheryl polyethylene glycol 1000 succinate (TPGS), cholesterol, lipopolysaccharides (LPS), 3-(4,5-dimethylthiazol-2-yl)-2,5-diphenyltetrazolium bromide (MTT), coumarin 6, pepsin, pancreatin, bile salt, and a BCA assay kit were obtained from Sigma Co. (St Louis, MO, USA). DiR (1,1′-dioctadecyl-3,3,3′,3′-tetramethylindotricarbocyanine iodide) was purchased from Invitrogen Molecular Probes (Karlsruhe, Germany). Eudragit^®^ S100 was donated by Evonik Korea Ltd. (Seoul, Korea). Dulbecco’s Modified Eagle’s medium (DMEM), Hank’s balanced salt solution (HBSS), non-essential amino acids, fetal bovine serum (FBS), penicillin–streptomycin, and all other reagents used in cell culture studies were obtained from GE Healthcare Life Sciences (South Logan, UT, USA). All other chemicals and reagents were HPLC-grade.

Caco-2 cells and RAW264.7 cells were purchased from the Korean Cell Line Bank (Seoul, Korea). Cells were grown in DMEM containing 10% FBS, 1% non-essential amino acids, and 1% antibiotics and incubated at 37 °C in an atmosphere of 5% CO_2_ and 90% relative humidity.

### Preparation of liposomes

Budesonide loaded-liposomes (Bud-Lip) were prepared by the thin film method. Briefly, DMPC, DMPG, Krill oil, TPGS, cholesterol, and drug (14:7:3:11:5:1, mole ratios) were dissolved in chloroform. For DiR-loaded liposomes, 0.5% DiR was included in the lipid mixture. After the lipids and the drug are thoroughly mixed in organic solvent, the solvent was removed by rotary evaporation above 25 °C and the resultant lipid film was dried overnight in a vacuum oven to remove the residual organic solvent. The resulting thin film was hydrated with 0.9% NaCl while stirring for 1 h. The mixture was, then, sonicated for 10 min. Finally, the liposome suspension was filtered through a membrane (0.8-μm pore size) to remove the free (unencapsulated) drug.

Aminoclay coated-liposomes (AC-Bud-Lip) were prepared via the electrostatic interactions between the positively charged aminoclay and the anionic surfaces of Bud-Lip. An aqueous solution of aminoclay (8 mg/mL) was mixed with an equal volume of Bud-Lip suspension (3 mg/mL) by stirring at 4 °C for 30 min. The resulting suspension was centrifuged through a membrane filter (MWCO = 30 kDa) at 15,000×*g* for 10 min at 4 °C. The pellets were re-suspended in 0.9% NaCl.

For Eudragit^®^ S100 coating, AC-Bud-Lip (3.5 mg/mL) was added drop-wise to an equal volume of Eudragit^®^ S100 (0.1% in 50 mM NaOH, pH 7.4) while stirring at 4 °C for 30 min. The resulting suspension was centrifuged through a membrane filter (MWCO = 30 kDa) at 15,000×*g* for 10 min at 4 °C. The pellets were re-suspended in 0.9% NaCl.

For the in vivo pharmacokinetic study in rats, budesonide was replaced by the hydrophobic fluorescent marker C-6 using the same procedures described above.

### Characterization of liposomes

The particle size and zeta potential of all liposomal formulations were measured by Dynamic Light Scattering using a Zetasizer (Nano-ZS90, Malvern Instruments, Malvern, UK). The polydispersity index (PDI) was also measured as a dimensionless number, which indicated the size distribution.

The encapsulation efficiency (EE %) of each formulation was calculated using the following equation: EE (%) = the entrapped drug amount in liposomes/the total drug amount initially added × 100. The drug amount was measured by high-performance liquid chromatography (HPLC).

The morphology of each formulation was examined by transmission electron microscopy (TEM). Liposomal samples were stained with 2% phosphotungstic acid and air-dried. Samples were monitored by TEM. Compositional elements of the developed formulations were also examined by using a TEM equipped with an Energy dispersive X-ray spectrometer (EDX).

Structural characterization of the liposomal formulations was also performed using Fourier transform infrared spectroscopy (FT-IR) (Nicolet™ iS™ 5; Thermo Fisher Scientific) with a ZnSe crystal accessory. FT-IR spectra of all samples were obtained over a wavenumber range of 4000–500 cm^−1^ with 64 scans at a resolution of 4 cm^−1^.

### In vitro drug release study

#### Drug release at different pHs

The in vitro drug release of liposomal formulations was evaluated at pH 1.2, 5.5, and 7.4. Each liposomal suspension (5 mL, equivalent to 3 μg/mL of budesonide) was added to a Spectra/Por (Spectrum Labs, CA, USA) dialysis bag (MWCO = 12–14 kDa), which was immersed in 20 mL of release medium containing 1% Tween 80 at 37 °C with horizontal shaking (100 rpm). At predetermined time points, 1 mL of each sample was collected and replenished by an equal volume of fresh medium. Samples were analyzed by HPLC to determine the released drug amount from each formulation.

Variation in the size of liposomal formulations was also evaluated during the incubation at the different pHs. Each formulation was suspended in buffer solutions (pH 1.2, pH 5.5, or pH 7.4) and incubated at 37 °C in a shaking water bath. At the predetermined time points, the sample was collected, and the particle size was measured. TEM and FT-IR analysis were also conducted to examine the structural and morphological changes during the incubation in each drug release medium.

#### Drug release in simulated gastrointestinal fluids

The in vitro drug release studies were carried out in the simulated gastric fluids (SGF) and intestinal fluids (SIF). SGF and SIF were prepared as described in the previous reports [26, 27] with slight modification, where SGF included pepsin (0.32 mg/mL) and SIF contained bile salt (0.5 mg/mL) and pancreatin (1 mg/mL). Each formulation dispersed in SGF or SIF (5 mL, equivalent to 3 μg/mL of budesonide) was placed into a Spectra/Por (Spectrum Labs, CA, USA) dialysis bag (MWCO = 12–14 kDa), which was immersed in 20 mL of release medium containing 1% Tween 80 at 37 °C with horizontal shaking (100 rpm). At predetermined time points, 1 mL of each sample was collected and replenished by an equal volume of fresh medium. Samples were analyzed by HPLC to determine the released drug amount from each formulation.

### Cellular uptake study

Caco-2 cells were seeded in 6-well plates at a density of 5 × 10^5^ cells/well. After 5-days post-seeding, the medium was removed, and the cells were washed twice with PBS. Cells were then incubated with each formulation (Bud solution, Bud-Lip, AC-Bud-Lip, and EAC-Bud-Lip) at the concentration equivalent of 15 μM Bud. The uptake studies were performed at pH 5.5 and pH 7.4. After 6 h of incubation, the drug solutions were removed and the cells were washed three times using ice-cold PBS. After cell lysis, acetonitrile was added to the cell lysates with vigorous stirring followed by centrifugation at 15,000×*g* for 15 min. The supernatants were collected and the drug concentration was determined by HPLC. Protein quantification for each sample was performed using a BCA protein assay kit.

### Confocal laser scanning microscopy (CLSM)

The intracellular localization of fluorescence-labeled liposomes was examined by confocal microscopy. Briefly, cells were seeded onto culture slides at a density of 1 × 10^5^ cells/well and incubated for 24 h. After removing the medium, 1 μM of bodipy-tagged liposomes were added, and the cells were incubated for 6 h. At the end of incubation, the cells were fixed with 4% paraformaldehyde in PBS for 15 min at 4 °C and then washed three times with PBS. Fixed cells were incubated with blocking buffer (1% horse serum and 0.1% Triton X-100 in PBS) for 30 min at 4 °C, and their nuclei were stained with DAPI for 1 h at 4 °C. Images of the intracellular distribution of liposomes were obtained by Nikon C1 confocal microscope and processed with EZ-C1 software (Nikon, Tokyo, Japan).

### Assessment of anti-inflammatory activity

The suppression effect of liposomal formulations on the production of proinflammatory cytokines (IL-6 and TNF-α) was evaluated and compared with free drug solution. Raw264.7 macrophages were seeded in 96-well plates at a density of 5 × 10^4^ cells/well. After incubation for 24 h, cells were treated with each formulation (free drug solution, Bud-Lip, AC-Bud-Lip and EAC-Bud-Lip) at a concentration equivalent to 10 μM Bud for 3 h and then stimulated with LPS (0.1 μg/mL) for 3 h. At the end of incubation, the extracellular media was collected. The amounts of TNF-α and IL-6 in each sample were determined by using their respective enzyme-linked immunosorbent assay (ELISA) kits (Thermo Fisher Scientific, Waltham, MA) according to the manufacturer’s instructions.

The cytotoxicity of each formulation on macrophages was also assessed by using an MTT assay. Raw264.7 macrophages were seeded in a 96-well plate and after 24 h-incubation, cells were treated with each formulation at various concentrations for 4 h. At the end of incubation, 50 μL of MTT was added into each well and incubated for another 4 h. Subsequently, the medium was removed and 100 μL of DMSO was added to dissolve the formazan crystals. The absorbance of each sample was determined by a microplate reader at 550 nm.

### Ex-vivo imaging study

The gastrointestinal distribution of three different formulations was evaluated in mice by using an in vivo imaging system (IVIS) (FOBI; Neoscience, Suwon, Republic of Korea). The near-infrared dye DiR was loaded as a fluorescent probe into each liposomal formulation.

Animal studies were carried out following the study protocol approved by the review committee of Dongguk University (IACUC-2017-016-1). Male C57BL/6 mice (8 weeks of age, body weight 23–28 g) were purchased from Orient Bio Inc. (Seongnam, Korea). All animals were allowed free access to water and were fasted for 24 h prior to the experiment. On the day of the experiment, DiR-loaded liposomes were administered orally to mice at a dose equivalent to 50 μg/kg of DiR. Blank liposomes (without DiR) were also tested. At predetermined time points (6, 12 and 24 h), the whole gastrointestinal tract of each mouse was isolated and the fluorescence signals were assessed by Spectral Lago X system (Spectral Instruments Imaging; Tucson, AZ, USA). Fluorescence images were obtained using an ICG filter at the excitation and emission wavelengths of 745 nm and 800 nm, respectively.

### Pharmacokinetic study

This animal study was carried out following the protocol approved by the review committee of Dongguk University (IACUC-2017-016-3). Male Sprague Dawley rats of 220–250 g were purchased from Samtako Bio Co., Ltd (Osan, Korea). All rats were given free access to tap water and a normal standard chow diet (Superfeed Co., Wonju, Korea). Rats were fasted for 18 h before the experiments. On the day of the experiment, the rats were divided into four groups and administered each C6-loaded formulation or free C6 solution (0.2 mg/kg) via oral gavage. Blood samples were collected at predetermined time points (0, 0.5, 1, 2, 4, 6, 8, and 24 h) and centrifuged immediately at 13,000×*g* for 5 min. The obtained plasma samples were stored at − 80 °C until analyzed. Colons were also resected at 24 h post-dose, and rinsed with cold saline to remove the luminal content. The colon samples underwent confocal microscopic examination and C6 content was quantified. The C-6 contents in the plasma and colon samples were extracted with a chloroform/methanol (1:1 v/v) mixture and quantified with a fluorescence plate reader (SpectraMax M2, Molecular Devices, LCC., San Jose, CA).

### HPLC analysis

Drug concentrations were determined by HPLC. The HPLC system (Flexar; Perkin Elmer, Waltham, MA, USA) consisted of an automatic injector, a UV detector and two solvent delivery pumps. Samples were injected into the HPLC system connected to a column (Gemini C18, 4.6 × 150 mm, 5 μm; Phenomenex, Torrance, CA, USA). Chromatographic separation was achieved by eluting a mobile phase (methanol: 0.1% acetic acid = 73:27, v/ v) at a flow rate of 1 mL/min. The detection wavelength was set at 245 nm. The calibration curve was obtained in the range of 125–5000 ng/mL with good linearity (R^2^ > 0.998).

### Pharmacokinetic analysis and statistical analysis

Noncompartmental analysis was performed and the area under the plasma concentration–time curve (AUC) was calculated using the linear trapezoidal method. The peak plasma concentration (C_max_) and the time of reaching the peak plasma concentration (T_max_) were observed from the experimental data.

All data are expressed as mean ± standard deviation (SD). Statistical analyses were performed using one-way ANOVA followed by Dunnett’s test. Values of *p* < 0.05 were considered statistically significant.
